# A systematic review of clinical practice guidelines for myopic macular degeneration

**DOI:** 10.7189/jogh.12.04026

**Published:** 2022-03-26

**Authors:** Yanxian Chen, Xiaotong Han, Iris Gordon, Sare Safi, Gareth Lingham, Jennifer Evans, Jinying Li, Mingguang He, Stuart Keel

**Affiliations:** 1 Department of Ophthalmology, Peking University Shenzhen Hospital, Shenzhen Peking University-The Hong Kong University of Science and Technology Medical Center, Shenzhen, China; 2 State Key Laboratory of Ophthalmology, Zhongshan Ophthalmic Center, Sun Yat-sen University, Guangdong Provincial Key Laboratory of Ophthalmology and Visual Science, Guangzhou, China; 3 Cochrane Eyes and Vision, International Centre for Eye Health, London School of Hygiene and Tropical Medicine, London, UK; 4 Ophthalmic Epidemiology Research Center, Shahid Beheshti University of Medical Sciences, Tehran, Iran; 5 Centre for Ophthalmology and Visual Science, Lions Eye Institute, University of Western Australia, Perth, Australia; 6 Centre for Eye Research Australia; Ophthalmology, Department of Surgery, University of Melbourne, Melbourne, Australia; 7 Vision and Blindness Prevention Programme, World Health Organization, Geneva, Switzerland

## Abstract

**Background:**

Myopic macular degeneration (MMD) is a primary cause of blindness and visual impairment in many parts of the world. A review of clinical practice guidelines (CPGs) for intervention selection are required with the increasing demand for MMD management in clinical practice as well as in national health services. Therefore, we aim to systematically review CPGs for MMD and assist the recommendations development of the Package of Eye Care Interventions (PECI) program of the World Health Organization.

**Methods:**

A systematic review of CPGs published on MMD between 2010 and April 2020 was conducted. Guidelines were evaluated using the Appraisal of Guidelines for Research and Evaluation II (AGREE II) tool. Cochrane systematic reviews were also included when the evidence from included CPGs were inadequate or contradict.

**Results:**

After applying exclusion criteria and conducting the quality appraisal, two CPGs were finally included. The average of the AGREE II ratings for the identified Guidelines were 56 and 63 respectively (7 for each item). To provide further information on interventions for MMD, one Cochrane review on MMD was additionally identified and included in the study. Intravitreal anti-vascular endothelial growth factor (anti-VEGF) drugs were recommended for patients with myopic choroidal neovascularization (mCNV) as first-line therapy to improve vision and reduce central macular thickness, and ranibizumab showed significant effectiveness compared to photodynamic therapy (PDT). PDT was recommended to be performed in those resistant to the treatment by one CPG but lacked of adequate description and support. Data extracted from the Cochrane systematic reviews indicated that anti-VEGF therapy for mCNV had significant effectiveness in improving visual acuity and reducing CMT compared to PDT with moderate to low certainty of evidence. Ranibizumab and bevacizumab were considered as equally effective with moderate certainty.

**Conclusions:**

The outcomes of this review suggest that high quality clinical practice guidelines for MMD management are limited. Intravitreal injection of anti-VEGF agents was recommended as an effective intervention to treat myopic CNV as the first-line treatment, while there was inadequate guidance for the application of PDT in myopic CNV management. The use of other interventions for MMD were not recommended at this time and additional evidence is called for.

Myopic macular degeneration (MMD), also known as degenerative myopia or pathological myopia, is an important cause of blindness and visual impairment in many parts of the world, especially in areas with a high prevalence of myopia [1,2]. It is characterized by the presence of staphyloma, lacquer cracks, Fuchs’ spot or chorio-retinal atrophy at the posterior pole. The prevalence of MMD is reported to be 0.9%-3.1% amongst adults aged 30 years and above in Asia,[3-5] 1.2% in European populations older than 49 years,[6] and can be as high as 12.0% in those aged >70 years.[7] A rapid rise of this age-related condition can be anticipated in the near future when the young population with a much higher rate of high myopia grow older,[8-11] posing a profound impact on the public health and eye care service.

Various treatment strategies for MMD have been explored in the last few decades, including macular surgical management,[12-14] laser photocoagulation for subfoveal or extra-foveal choroidal neoucascularization,[15,16] photodynamic therapy (PDT),[17-19] corticosteroid treatment [20,21] and anti-vascular endothelial growth factor (anti-VEGF) therapy.[22-26] Though data have shown some of these treatments are promising, such as anti-VEGF agents [27], the cost utility of these treatments have not been consistently estimated for the purpose of guiding decision-making process of countries and national health services. A systematic review to find the priority of intervention selection for MMD will be of great value.

Given the increasing need for eye care, the World Health Organization (WHO) has highlighted the role of eye care in contributing to the Sustainable Development Goals (SDGs), and is developing an evidence-based Package of Eye Care Interventions (PECI). The methodology for the development of the PECI has been previously published.[28] In brief, the PECI aims at providing a systematic identification of evidence-based eye care interventions for pre-defined priority eye conditions based on high quality clinical practice guidelines (CPGs) and, where needed, systematic reviews, to assist the choice of interventions in clinical decision-making and national health services. This paper aims to present the results of a systematic review of CPGs for MMD, including the quality and current state of evidence, as a part of PECI development.

## METHODS

The systematic review of CPGs is following the procedures reported in the methodology paper previously [28]. Exclusion criteria for each stage of screening can be seen in **Table 1**. The stages in the review are as follows:

**Table 1:** Exclusion criteria in the screening process of clinical practice guidelines

Title & Abstract screening
1) The identified literature was not a CPG
2) The guideline was not published in the last 10 y
3) The guideline was not in English
4) The guideline was not developed for the selected eye conditions
Full text screening:
1) There was commercial funding or unmanaged conflicts of interest present
2) Absence of affiliation of authors
3) The guideline was not developed for the selected eye conditions
Quality Appraisal:
1) The average score of the two researchers for items 4, 7, 8, 12, or 22 is below 3
2) the sum of the average score of the two researchers for all nine items is less than 45

CPG – clinical practice guideline

### Systematic literature search

A systematic literature search of academic databases (MEDLINE, Embase, CINAHL, Global Health, Global Index Medicus) and guideline databases (as listed in Appendix S1 of the **Online Supplementary Document**) was carried out by an information specialist from Cochrane Eyes and Vision (CEV). In addition, professional ophthalmology and optometry associations’ websites were searched for relevant guidelines (Appendix S1 on the **Online Supplementary Document**). We restricted the searches to the last 10 years and to English language. The search strategy for the academic databases can be found in Appendix S2 on the **Online Supplementary Document**.

### Literature screening and quality appraisal

All the titles and abstracts of articles identified from the literature searches were screened independently by two authors (G.L and S.S). Abstrackr, a semi-automated online citation screening program, was utilized where possible [29]. Concerns about the eligibility were discussed with a CEV representative (J.E.) and the WHO representative (S.K). Two authors (Y.C and X.H) conducted full-text screening of the CPGs relevant to MMD independently, and discussed with a third author when there were disagreements.

Two authors (Y.C and X.H) independently evaluate the quality of the included CPGs using “Appraisal of Guidelines for Research and Evaluation” (AGREE II) [30]. Items 4, 7, 8, 10, 12, 13, 15, 22 and 23 in AGREE II (Appendix 3 on the **Online Supplementary Document**) were specifically selected according to a consensus finding process [31]. A scale of importance from strongly disagree (1 point) to strongly agree (7 point) was used in each item. A difference of more than 2 points for any item between the two authors were discussed between the two authors to reach a consensus, and the representative of WHO or CEV was involved when necessary.

### Guideline selection and data extraction

Following evaluation with the AGREE II tool, guidelines were excluded if the average rating of the two authors for items 4, 7, 8, 12 or 22 was less than 3, or the total rating for all 9 items was below 45. Final selection of a maximum of 5 CPGs according to the following criteria: 1) quality 2) publication time and 3) comprehensiveness (ie, applicability to different settings) with the agreement of the whole study group.

Information related to the recommendation, including type of recommendation, dosage, target population, the strength of recommendation and the quality of the evidence supporting the recommendation, were extracted by one author (Y.C or X.H) and re-reviewed by another author. Disagreements were discussed and resolved by the two researchers and a third author (S.K.). The process was repeated for all the Guidelines until agreement on the recommended eye care interventions was reached.

With respect to the published protocol no changes were performed. The quality check and the methodological support for this study have been provided by WHO/CEV.

### Search and selection of systematic reviews

When the evidence from included CPGs were inadequate or contradict, Cochrane Systematic Reviews (CSRs) were sought. The Information Specialist for CEV identified CSRs published in the last 10 years by searching the Cochrane library using the term “myopic macular degeneration. The following search limits were defined for the search strategy in the Cochrane library: 1) Content type: Cochrane Reviews, 2) Cochrane Library publication date: last 10 years, 3) Search word variations: Yes. The search was run on 23 July 2020 and retrieved 7 CSRs. The CEV Information Specialist pre-screened the results and forwarded PDFs of two CSRs that were potentially relevant for inclusion in the review.

Two members of the group (Y.C and X.H) independently performed title and abstract screening. Systematic reviews were excluded if they were older than 10 years or the review was not an intervention or diagnostic test accuracy review that was specifically related to the target eye condition. After comparing the decisions of both researchers, only those CSRs where there was agreement between both authors (Y.C and X.H) were included.

## RESULTS

**Figure 1:**
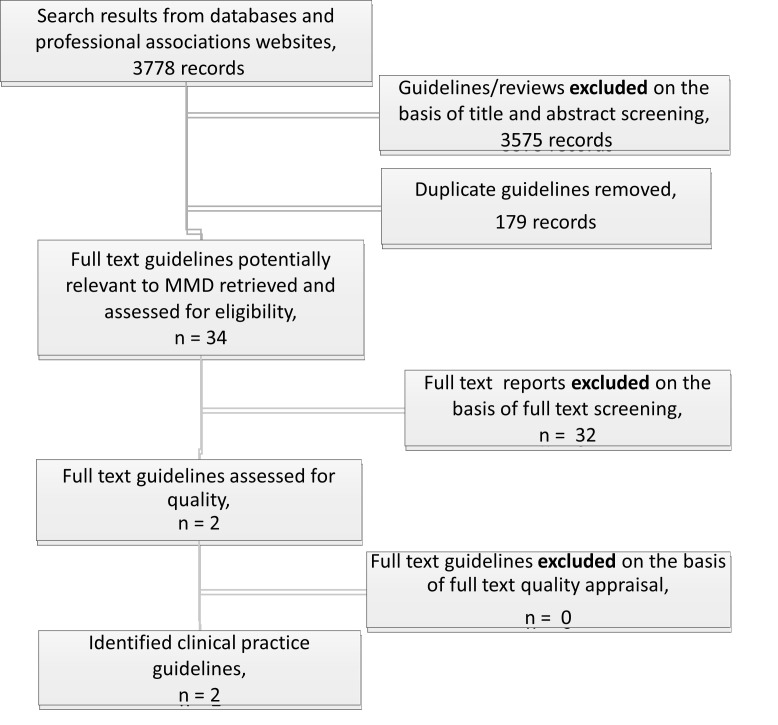
Flowchart for the results of the screening process. CPG: clinical practice guideline.

After combining all searches from academic and guideline databases and professional association webpages, 3778 CPGs were identified and underwent independent title and abstract screening. A total of 3575 CPGs met the exclusion criteria and 179 were duplicates, leaving a total of 34 CPGs that were considered potentially relevant and were retrieved to undergo full-text screening and quality appraisal. Of the identified 34 guidelines, we excluded 32 Guidelines for the following reasons: 29 because the content was not relevant to MMD after full-text screening [32-60], 3 because the absence or presence of possible conflicts of interest was not stated [27,61,62] (**Figure 1**).

After conducting quality appraisal on the remaining CPGs, and checking for quality, publication time and comprehensiveness, we finally included the following guidelines: “Intravitreal injection of anti-vascular endothelial growth factor agents for ocular vascular diseases; Clinical practice guideline”(2018) [63] developed by Homayoun Nikkhah et al., and “Ranibizumab for treating choroidal neovascularisation associated with pathological myopia”(2013) [64] developed by National Institute for Health and Care Excellence (NICE). The total AGREE II ratings for the former guideline were 56, and this figure for NICE guideline were 63, both were above 45 points (**Table 2**).

**Table 2:** The AGREE II rating of the selected guidelines after full-text screening

Guideline		AGREE II ratings						Topic	Publication Date (Y/N)	Comprehensiveness (applicability to different settings)
**Total**	**Average of key items**					
	4	7	8	12	22	4,7,8,10,12,13,15,22,23
Intravitreal injection of anti-vascular endothelial growth factor agents for ocular vascular diseases; Clinical practice guideline	56	7	5	4	7	7	6.2	Myopic choroidal neovascularization	2018	Applicable to clinical interventional procedures
Ranibizumab for treating choroidal neovascularisation associated with pathological myopia	63	7	7	7	7	7	7	choroidal neovascularization associated with pathological myopia	2013	Applicable to clinical interventional procedures

MMD – myopic macular degeneration

The included CPG developed by Nikkhah et al. recommended intravitreal anti-VEGF drugs for patients with myopic choroidal neovascularization (mCNV) to improve vision and to reduce central macular thickness. Intravitreal bevacizumab was recommended to be the first-line injection for these patients, and photodynamic therapy (PDT) performed in those resistant to the treatment. The evidence for the recommendations came from 1 meta-analysis, 1 RCT and 1 review, and the evidence level was graded as high. The CPG also recommended that highly myopic patients aged less than 50 years should have funduscopy to ensure early detection of MMD. For older patients with risk factors of CNV recurrence (high degrees of myopia and subfoveal CNV, primary extensive CNV, hemorrhage, choroidal thickness reduction etc), periodic examinations are recommended, but the evidence level of this recommendation was low since it was only supported by case series. In the NICE CPG, intravitreal ranibizumab is recommended as an option for mCNV, and concluded that ranibizumab is a treatment with clinical effectiveness for visual impairment caused by mCNV, supported by 3 RCTs. But the long-term improvement (after 3 months) in BCVA brought by ranibizumab is uncertain. The recommendations and quality of evidence are summarized in **Table 3**.

**Table 3:** Recommended eye interventions for myopic macular degeneration and strength of recommendation and quality of evidence in selected clinical practice guidelines

Description of intervention	Relevant guideline*	Main comment	Quality of evidence
Intravitreal injection of anti-VEGF agent	1,2	It is recommended that intravitreal anti-VEGF drugs be used in patients with myopic CNV to improve the vision and to reduce CMT.	RCT, meta-analysis, review
		Ranibizumab is recommended as an option for treating choroidal neovascularisation associated with pathological myopia when the manufacturer provides ranibizumab with the discount agreed in the patient access scheme.	
Photodynamic therapy	1	In patients with myopic CNV, it is recommended that IVB be injected first, and photodynamic therapy should then be performed in cases resistant to the treatment.	Unclear†

*1: Intravitreal injection of anti-vascular endothelial growth factor agents for ocular vascular diseases; Clinical practice, 2: Ranibizumab for treating choroidal neovascularisation associated with pathological myopia.

†The listed references in the guideline have no information about the use of photodynamic therapy in resistant cases.

To complement the evidence from the CPG, all Cochrane reviews on the topic area were also checked. In total one Cochrane review on MMD was identified, “Anti-vascular endothelial growth factor for choroidal neovascularisation in people with pathological myopia (Review)” (2016) [65]. In the identified anti-VEGF review, anti-VEGF therapy was found to not cause significantly higher risk of systemic serious adverse events or ocular adverse events compared to PDT. Anti-VEGF therapy also showed a higher quality of life in MMD patients supported with moderate-certainty evidence, and was suggested to be significantly effective in improving visual acuity and reducing central macular thickness compared to PDT with moderate to low certainty of evidence (**Table 4**). Ranibizumab and bevacizumab were considered to be equally effective with moderate certainty.

**Table4:** Summary of interventions for myopic macular degeneration and certainty of evidence from identified Cochrane systemic reviews

Intervention name	Comparator intervention	Dosage/ frequency of the intervention	Outcomes	Relative effect (95% CI)	Certainty of evidence
Anti-vascular endothelial growth factor therapy	Photodynamic therapy	Ranibizumab 0.5 mg, Bevacizumab 1.25 mg, Aflibercept 2.0 mg. Frequency: 3+PRN or 1+PRN	Mean change from baseline in BCVA at 1 y after treatment.	-0.14logMAR (-0.20,-0.08)	Low
Anti-vascular endothelial growth factor therapy	Photodynamic therapy	Ranibizumab 0.5 mg, Bevacizumab 1.25 mg, Aflibercept 2.0 mg. Frequency: 3+PRN or 1+PRN	Proportion of participants with a gain of 3+ lines in BCVA at 1 y after treatment.	RR 1.86 (1.27,2.73)	Moderate
Anti-vascular endothelial growth factor therapy	Photodynamic therapy	Ranibizumab 0.5 mg, Bevacizumab 1.25 mg, Aflibercept 2.0 mg. Frequency: 3+PRN or 1+PRN	Change in central macular thickness at 1 y	17.84μm (6.3,41.98)	Moderate
Anti-vascular endothelial growth factor therapy	Photodynamic therapy	Ranibizumab 0.5 mg, Bevacizumab 1.25 mg, Aflibercept 2.0 mg. Frequency: 3+PRN or 1+PRN	CNV angiographic	RR 1.24 (0.99,1.54)	Low

BCVA – best corrected visual acuity, CI – confidence interval, PRN – pro re nata, RR – relative risk, CNV – choroidal neovascularization, CI – confidence interval

An overall view of the strength of recommendation and quality of evidence given in each Guideline is reported in **Table 4**. There are substantial evidence supporting intravitreal injection of anti-VEGF agent as a beneficial treatment of mCNV, and PDT is also recommended though with lower effectiveness. Laser photocoagulation is not strongly recommended since its role in treating non-subfoveal mCNV is yet to be identified.

## DISCUSSION

The current study reviewed CPGs and Cochrane systemic reviews for MMD, with the aim of providing evidence on eye care interventions for MMD to aid in the development of the PECI. A total of 2 CPGs and 1 Cochrane systemic review were included after a standard screening process. Anti-VEGF therapy was recommended to improve the vision in patients with myopic CNV, among which ranibizumab showed greater effectiveness compared with PDT, while PDT was suggested to use in resistant cases with no solid evidence. Overall, current high-quality CPGs and meta-analysis can provide some guidance for the management of myopic CNV, but the types of interventions involved are limited.

In recent years, anti-VEGF agents have been introduced and rapidly become an important therapy in the treatment of ocular vascular diseases, including age-related macular degeneration, diabetic macular edema, retinal vein occlusion and mCNV [22-24,66-70]. Anti-VEGF therapy is also a strongly recommended intervention for mCNV in the current study with significantly better visual outcome compared to PDT supported by moderate-certainty evidence [65]. Among the available anti-VEGF agents, ranibizumab and bevacizumab are believed to have equal treatment effect and similar long-term outcome for mCNV. The improved vision of mCNV patients after ranibizumab or bevacizumab intraocular injection was reported to be maintained up to 3 to 4 years [25,71-73], however the long term effects remain unclear [74]. While the clinical effectiveness of aflibercept for mCNV still needs to be confirmed with further comparisons with other agents and analysis of cost and utility, as well as long-term observation after treatment. Pegaptanib was reported to be effective in the treatment of mCNV in a case-series study and a case report [75,76], but its effectiveness needs evaluating in larger RCTs. Though anti-VEGF therapy is recommended as first-line therapy, it’s still unclear how long these agents can be used out of local and systemic safety concerns, and what should be done when the first treatment fails. Future guidelines should be developed aiming to fill these gaps.

Among the available treatments for mCNV, PDT with verteporfin has a long history as an established and approved treatment and has been shown to achieve satisfying short-term outcome in improving vision of mCNV patients [17-19,77-81]. However, the long-term visual outcome with PDT is controversial. Some case-series showed improved vision in mCNV patients after PDT that was maintained up to 2 to 3 years [17,82-86], while a randomized clinical trial reported that PDT did not have better visual outcomes compared to placebo after 2 years [87]. In addition, standard-fluence PDT may contribute to chorioretinal atrophy in long term [88,89]. In this study, the CPG from Nikkhah et al. recommended PDT to be performed in cases resistant to intravitreal anti-VEGF treatment, but we found no information about this regimen in the references they listed. The recommendation of PDT was more like a presumptive conclusion from the technical committee. It is also worth noticing that the cost of PDT is quite high, which is unfordable in less-developed areas. Therefore, the application of PDT as a major treatment option or an alternative therapy for myopic patients in clinical practice still needs to be validated with further cost and benefit analysis.

Despite the increasing demands for clinical management of MMD and other complications of pathological myopia, this study found few high-quality CPG. The CPG developed by Nikkhah et al provided clinical recommendations for 5 different ocular vascular diseases and evidence levels of each intervention, but presented a lack of details in terms of clinical effectiveness, cost-effectiveness, and technology of implementation. NICE, as a large government agency that develops guidelines, has only one comprehensive CPG for MMD. Though one Cochrane systemic review has been added to the current study, in total only recommendations on, and evidence for, two interventions were identified. The amount of benefit with surgical technology, including submacular surgery or macular translocation for mCNV, corticosteroid injection and combined therapy remains unclear. In an excluded CPG written by Cheung et al, a detailed recommendation of interventions were provided, that anti-VEGF therapy was considered as the first-line treatment with Class I recommendation, vPDT and intravitreal TA+ vPDT were Class IIa recommendation, macular surgery, laser photocoagulation and intravitreal TA + anti-VEGF therapy were Class IIb recommendation [61]. Our main conclusion that anti-VEGF therapy as first choice for mCNV was in consistence with these recommendations, confirming the status of anti-VEGF therapy among mCNV treatments. However, the types of interventions were limited in our review after excluding the CPGs evaluating more interventions, may lead to inadequate guidance on alternative interventions for patients with specific conditions, including multiple foci of mCNV [90], pregnant women [91] or ocular hypertension [92]. Institutes independent from manufacturers are expected to investigate all the available treatment options for MMD in the future to provide comprehensive guidance for MMD treatments.

There are some inherent limitations in this study. Based on the criteria for identifying guidelines to inform the development of the PECI, only two guidelines were included. Though there have been enough evidence supporting ophthalmologists to use anti-VEGF as first-line therapy for mCNV, guidance is still insufficient for those who have contradictions, specific conditions or failure in taking anti-VEGF therapy. Second, MMD is most prevalent in countries where English is not the primary language. Given the language barrier in publications, some guidelines for MMD may have been omitted in the current analysis. Future study with larger searching database are required to provide better-quality data to help establish the best management strategy for MMD. Third, to assure the objectivity of the results, three papers providing assessment on more than one intervention for MMD were excluded due to possible conflicts of interest. The efficacy and utility of alternative treatments could not be assessed adequately.

## CONCLUSIONS

There are limited clinical practice guidelines for MMD management met the inclusion criteria of PECI development. Based on existing evidence, intravitreal injection of anti-VEGF agents is suggested to be an effective therapy for myopic CNV and should be the first-line treatment. The application of PDT for mCNV in clinical practice is still lack of adequate guidance. The use of other interventions for MMD is not recommended at this time and further solid evidence is called for.

## Additional material


Online Supplementary Document
