# Supplementary material for: A systematic review of clinical practice guidelines for myopic macular degeneration
Source: J Glob Health. 2022 Mar 26;12:04026. doi: 10.7189/jogh.12.04026 (PMC8939288; doi:10.7189/jogh.12.04026)
Supplement: Online Supplementary Document [file jogh-12-04026-s001.pdf]

Appendixes

Appendix S1

Guideline databases

1. Guidelines International Network
2. National Institute for Clinical Excellence (UK)
3. eGuidelines
4. Guideline Central
5. ECRI Guidelines Trust Database
6. Australian National Health and Medical Research Council clinical practice guidelines
7. Canadian Medical Association Infobase of Clinical Practice Guidelines
8. Trip Database
9. WHO guidelines
10. US Preventive Services Task Force Guideline
11. Scottish Intercollegiate Guidelines Network (SIGN)

Professional association websites

1. College of Optometrists (UK)
2. Pan American Association of Ophthalmology
3. Eastern Mediterranean Council of Optometry
4. European Society of Ophthalmology
5. World Council of Optometry
6. American Academy of Ophthalmology
7. American Optometric Association
8. Asia Pacific Council of Optometry
9. International Council of Ophthalmology
10. Royal Australian and New Zealand College of Ophthalmologists
11. Royal College of Ophthalmologists
12. European Society of Cataract and Refractive Surgeons
13. American Society of Cataract and Refractive Surgeons
14. European Vitreoretinal Society
15. African Ophthalmology Council
16. Asia Pacific Academy of Ophthalmology

Appendix S2

Search strategy for academic databases

MEDLINE

1 exp eye diseases/ (557476)
2 visually impaired persons/ (2391)
3 or/1-2 (558065)
4 Practice Guidelines as Topic/ (115227)
5 Guidelines as Topic/ (39163)
6 Practice Guideline/ (26462)
7 ((clinical or practice) adj2 guideline$).tw. (41838)
8 or/4-7 (202728)
9 3 and 8 (2180)
10 exp case reports/ (2078744)
11 case report$.tw. (356889)
12 or/10-11 (2160660)
13 9 not 12 (2112)
14 limit 13 to english language (1676)
15 limit 14 to yr="2009 -Current" (949)
16 limit 15 to (address or autobiography or bibliography or biography or classical article or clinical trial, veterinary or clinical trials, veterinary as topic or comment or directory or editorial or "expression of concern" or festschrift or historical article or interactive tutorial or interview or letter or news or newspaper article or observational study, veterinary or personal narrative or portrait or video-audio media or webcast) (113)
17 15 not 16 (836)

Embase

1 exp eye disease/cn, di, dm, ep, et, pc, rh, su, th [Congenital Disorder, Diagnosis, Disease Management, Epidemiology, Etiology, Prevention, Rehabilitation, Surgery, Therapy] (314642)
2 visual impairment/cn, di, dm, ep, et, pc, rh, su, th [Congenital Disorder, Diagnosis, Disease Management, Epidemiology, Etiology, Prevention, Rehabilitation, Surgery, Therapy] (6819)
3 or/1-2 (314642)
4 practice guidelines/ (405820)
5 ((clinical or practice) adj2 guideline$).tw. (60447)

6 or/4-5 (428306)
7 3 and 6 (3082)
8 exp case report/ (2347870)
9 case report$.tw. (464572)
10 or/8-9 (2402846)
11 7 not 10 (2932)
12 limit 11 to english language (2574)
13 limit 12 to yr="2009 -Current" (1346)
14 limit 13 to (conference abstract or conference paper or editorial or letter or note or short survey or tombstone) (257)
15 13 not 14 (1089)

Global Health

1 exp eye diseases/ (32535)
2 people with visual impairment/ (170)
3 or/1-2 (32658)
4 exp guideline/ (47207)
5 ((clinical or practice) adj2 guideline$).tw. (4484)
6 or/4-5 (49229)
7 3 and 6 (333)
8 case reports/ (86862)
9 case report$.tw. (94202)
10 8 or 9 (94202)
11 7 not 10 (329)
12 limit 11 to english language (279)
13 limit 12 to yr="2009 -Current" (197)

CINAHL

S5 S1 AND S4 Limiters - Published Date: 20090101-20200231; English Language; Exclude MEDLINE records
S4 S2 OR S3
S3 (TX clinical OR TX practice) N2 (guideline*)
S2 MM "Practice Guidelines"
S1 MH "Eye Diseases+"

WHO Global Index Medicus

SEARCH 1

(ab:(practice guideline)) AND (ab:(eye OR vision OR visual OR blindness))

SEARCH 2

(mh:(eye diseases)) AND (mh:(practice guideline))

Appendix S3

Items selected from APPRAISAL OF GUIDELINES fOR RESEARCH & EVALUATION II:

Domain 2. Stakeholder involvement

Item 4. The guideline development group includes individuals from all the relevant professional groups.

Domain 3. Rigour of development

Item 7. Systematic methods were used to search for evidence.

Item 8. The criteria for selecting the evidence are clearly described.

Item 10. The methods for formulating the recommendations are clearly described.

Item 12. There is an explicit link between the recommendations and the supporting evidence.

Item 13. The guideline has been externally reviewed by experts prior to its publication.

Domain 4. Clarity of presentation

Item 15. The recommendations are specifc and unambiguous.

Domain 6. Editorial independence

Item 22. The views of the funding body have not influenced the content of the guideline.

Item 23. Competing interests of guideline development group members have been recorded and addressed.
